# Neurobiological and Pharmacological Perspectives of D3 Receptors in Parkinson’s Disease

**DOI:** 10.3390/biom12020243

**Published:** 2022-02-01

**Authors:** Abdeslam Chagraoui, Giuseppe Di Giovanni, Philippe De Deurwaerdère

**Affiliations:** 1 Différenciation et Communication Neuroendocrine, Endocrine et Germinale Laboratory, Institute for Research and Innovation in Biomedicine of Normandy (IRIB), University of Rouen, INSERM 1239, 76000 Rouen, France; 2 Department of Medical Biochemistry, Rouen University Hospital, 76000 Rouen, France; 3 Laboratory of Neurophysiology, Department of Physiology and Biochemistry, Faculty of Medicine and Surgery, University of Malta, 2080 Msida, Malta; giuseppe.digiovanni@um.edu.mt; 4 Neuroscience Division, School of Biosciences, Cardiff University, Cardiff CF10 3AT, UK; 5 Unité Mixte de Recherche (UMR) 5287, Centre National de la Recherche Scientifique (CNRS), CEDEX, 33000 Bordeaux, France; philippe.de-deurwaerdere@u-bordeaux.fr

**Keywords:** DA replacement therapy, D1R–D3R heteromer, l-DOPA, pharmacology, basal ganglia, neurobiology, dyskinesias

## Abstract

The discovery of the D3 receptor (D3R) subtypes of dopamine (DA) has generated an understandable increase in interest in the field of neurological diseases, especially Parkinson’s disease (PD). Indeed, although DA replacement therapy with l-DOPA has provided an effective treatment for patients with PD, it is responsible for invalidating abnormal involuntary movements, known as L-DOPA-induced dyskinesia, which constitutes a serious limitation of the use of this therapy. Of particular interest is the finding that chronic l-DOPA treatment can trigger the expression of D1R–D3R heteromeric interactions in the dorsal striatum. The D3R is expressed in various tissues of the central nervous system, including the striatum. Compelling research has focused on striatal D3Rs in the context of PD and motor side effects, including dyskinesia, occurring with DA replacement therapy. Therefore, this review will briefly describe the basal ganglia (BG) and the DA transmission within these brain regions, before going into more detail with regard to the role of D3Rs in PD and their participation in the current treatments. Numerous studies have also highlighted specific interactions between D1Rs and D3Rs that could promote dyskinesia. Finally, this review will also address the possibility that D3Rs located outside of the BG may mediate some of the effects of DA replacement therapy.

## 1. Introduction

Dopamine (DA) D3 receptor (D3R) subtypes were discovered in 1990 by Jean-Charles Schwartz, Pierre Sokoloff, and colleagues [1]. Almost immediately, D3Rs attracted interest in the field of Parkinson’s disease (PD) to determine the extent to which these receptors mediated the benefits and side effects of DA replacement therapy encompassing the metabolic DA precursor l-DOPA or DAR agonists [2]. Nowadays, they are considered as a potential target in drug addiction, depression, restless leg syndrome, and schizophrenia [3,4]. For most diseases, their use is still mainly at the level of preclinical data. However, the targeting and blockade of D3R in newer antipsychotic drugs seems to be well acknowledged [4].

PD is a complex neurodegenerative disease marked by motor symptoms encompassing bradykinesia, rigidity, postural instability, and tremor at rest. Non-motor symptoms are also numerous, though they are not included in the clinical description [5]. The motor symptoms are attributed to the profound changes in the function of the basal ganglia (BG), a group of subcortical regions involved in the control of motor behavior, as a consequence of the degeneration of DA neurons of the substantia nigra (SN) innervating the striatum [6,7,8,9]. The destruction of DA neurons is insidious before the onset of the disease and continues at different rates after the diagnosis, according to the forms of the disease [10]. Parkinson’s disease is also associated with other alterations in groups of neurons, including noradrenergic, serotonergic, and mesencephalic cholinergic neurons, at various degrees. The damage to these different systems is likely to participate in the clinical outcomes of de novo patients and to the benefits and side effects of the current treatments for the disease [11,12,13,14,15,16,17,18,19,20,21,22].

The D3R is expressed in various tissues of the central nervous system (CNS), including the striatum. In the context of PD, the main interest has been devoted to striatal D3Rs and motor side effects, including dyskinesia, occurring with DA replacement therapy. Therefore, this review will briefly describe the BG and the transmission of DA within these brain regions, before going into more detail with regard to the role of D3Rs in PD and their participation in current treatments. Numerous studies have also highlighted specific interactions between D1Rs and D3Rs that could promote dyskinesia. Finally, the review will also address the possibility that D3R located outside of the BG could mediate some of the effects of DA replacement therapy.

## 2. Functional Anatomy of the Basal Ganglia in Parkinson’s Disease

**Figure 1:**
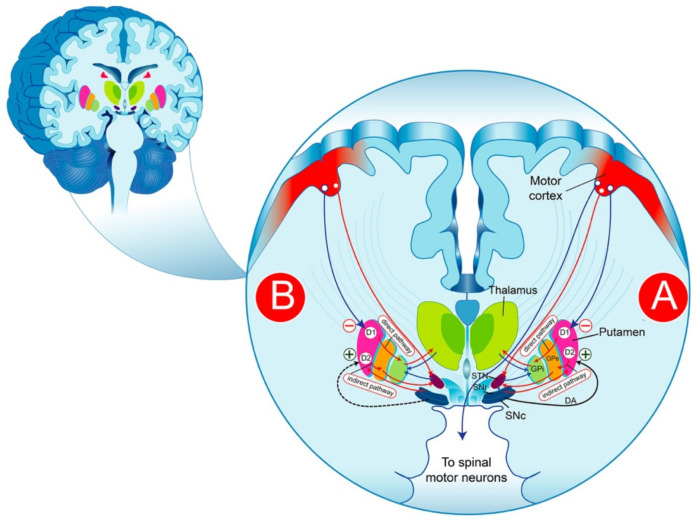
Schematic representation of DAergic pathways that regulate movement in the physiological condition (**A**) and PD (**B**). The substance P/dynorphin-positive MNS GABAergic neurons, expressing primarily D1R, send projections directly to the SNr and/or to the GPi. These neurons exert a tonic inhibition on the thalamic glutamatergic nucleus, which in turn sends excitatory projections to the motor cortex, thereby forming the direct pathway. The striatal enkephalin-positive MNS GABAergic neurons, expressing largely D2R, send projections to the GPe, which in turn project their axons to the glutamatergic neurons of the subthalamic nucleus and inhibit the neurons in this structure. In the subthalamic nucleus, the neurons are excitatory glutamatergic neurons that send projections to the globus pallidus (internal segment) resulting in the disinhibition of the subthalamic nucleus neurons, which, in turn, activate neurons in the internal segment of the globus pallidus to inhibit the thalamus, reflected by the inhibition of movements. DA-dopamine; GPi-globus pallidus internus; SNr-substantia nigra reticulata; SNc-nigra pars compacta; STN-subthalamic nucleus; GPe-globus pallidus pars externa; GPi-globus pallidus pars interna.

The BG are a group of structures that are connected via glutamatergic and GABAergic neurons. The striatum plays a central role in the functional anatomy of the BG, receiving glutamatergic cortical projections and contacting efferent systems via two distinct, parallel pathways: the direct and the indirect pathway. It is commonly thought that the reduction in the inhibitory activity of the “indirect pathway” producing a significant increase in the excitatory activity of the “direct pathways” on the substantia nigra reticulata (SNr)/GPi) neurons is likely the result of the degeneration of nigrostriatal cells, as expressed by a reduction in the striated DA (Figure 1). The pharmacological control of the symptoms of PD is thought to consist of re-establishing the basal balance between the direct and indirect pathways through DA precursors or DA receptor (DAR) agonists that promote DA transmission in the striatum. This model provides a partial explanation of some of the cardinal characteristics of PD and the pharmacological effectiveness of DA drugs. In fact, the interpretation of the pathophysiological processes becomes more complex than initially assumed and requires an adjustment of the classical BG model; thus, the question remains as to how to explain the progression of the disease.

There are two conflicting reports [23] regarding the information arising from various cortical regions or different somatotopic areas. One suggests that the information originating from different cortical regions is always considered independently in different regions of the BG [24,25,26]. According to another view, the BG (especially the striatum) achieves an integrative convergence of the cortical information [27]. Both views are thought to be likely to function; the integration of information is especially pertinent when originating from cortical regions exhibiting similar functions. Thus, the same striatal areas could receive projections from the primary motor cortex and the supplementary motor region, while cortical regions exhibiting different functions could project to different areas of the striatum. Changes in these positional relationships within the BG loops may be altered in PD. In addition, some structural features should be considered. Indeed, several short loops do exist in the BG model, including the SNc-striatum-SNc loop, the striatum-SNr-intralaminar nuclei of the thalamus–striatum loop, and the thalamo-cortex-thalamo glutamatergic loop [28]. Several structural features of the BG are not permanent, since they may be subject to changes as a result of various factors. One illustration of this is the induction of plasticity at the cortico-striatal synapse, associated with short-term and long-term changes modulated by glutamate and DA, therefore impacting motor capacities [29,30,31,32]. Furthermore, animal studies and clinical investigations have shown that a decrease in DA results in a significant reduction in the number of spines of medium spiny neurons (MSNs); these structural modifications could presumably impair motor functions [33,34].

## 3. The Complexity of the Dopaminergic Neuron Network

The classical model of the BG has proved to be very useful in better understanding of the issues related to the pathophysiology of PD during the earliest stage of this disease. However, its interpretative value has proven to be insufficient with disease progression. Nigrostriatal neurons were thought to be a homogeneous cell group, with the somata situated within the substantia nigra pars compacta (SNc) possessing synaptic projections solely innervating the striatum. This view has progressively evolved with a new robust body of evidence from numerous studies. In fact, a population of non-nigral mesencephalic DA neurons is distinguished by their projections to the striatum. Furthermore, DA neurons of the nigra can send projections to brain areas other than the striatum, including the globus pallidus, the subthalamic nucleus, several thalamic nuclei, and the cortex [35,36,37,38,39,40]. The DA cells of the nigra differ not only in their neurochemical characteristics but also in the proteins necessary for the synaptic management of DA and the expression of DA transporters of the cell membrane and synaptic vesicles [41,42]. Thus, DA cell groups in the nigra exhibit different expressions of calretinin, calbindin [43], and cholecystokinin [44], and also different accumulations of melanin [45,46,47]. These differences may explain, in part, the functional distinctions seen between nigral DA cells and presumably their heterogeneous vulnerability to internal (DA oxidation) or external factors [43,48].

Based on microdialysis studies, the key is to maintain an extracellularly stable level of DA in the striatum; thus, the therapeutic strategy consisted of replacing DA with drugs capable of simulating post-synaptic DARs. It should be kept in mind that DA may exert short, local effects that cannot be substituted by drugs. Furthermore, given the diversity of DA cells, the cellular response to drugs may differ from that induced by DA [49,50]. Indeed, microdialysis fails to explain the self-regulation process of DA cells, and their ability to integrate information originating from different brain areas. In addition, the partial degeneration of the DA system involves several regulatory mechanisms of DA synthesis, including presynaptic receptors (DA-release), intracellular calcium level, the modulation of tyrosine hydroxylase biological activity, DA uptake, and DA post-synaptic activity. These regulating mechanisms may be modulated differently depending on whether DA is released in the striatum or the SN [50,51,52].

In addition, as the disease progresses, the neurodegenerative process reaches other brain areas, including the amygdala, the hippocampus, and the limbic system, and extends to the cortical primary receptive areas and the cortical multi-modal association regions [53].

## 4. D3 Dopamine Receptor Expression in Parkinson’s Disease

It is widely agreed that the distribution of D3R is more restricted than that of D2R, with distinct densities [54]. It has been reported that the expression of D3R mRNA is limited to some brain regions, such as the nucleus accumbens, mainly rostral pole and shell subdivisions, the ventral pallidum, the islands of Calleja, and at lower levels in the striatum [1,55,56]. Nonetheless, it should be noted that the expression profile of D3R in rodents differs from that of the primate brain. In primates and humans, the whole striatum expresses D3Rs, with abundant levels in the nucleus accumbens [2,55,57,58,59]. The expression of D3Rs is also observed in the anterior thalamus, amygdala, cortex, hippocampus, and internal segment of the globus pallidus (GP) of primates, while expression is undetectable in rodents [55,59].

The pharmacological interest in the D3R lies in its high affinity for the endogenous agonist DA and most D2R agonists [1]. D2R/D3R are coupled to inhibitory Gαi/o proteins for intracellular signaling [60]. Beyond these canonical pathways, several intracellular partners may interact with the DARs, including beta-arrestin in addition to the regulator of G protein signaling (RGS) proteins [61]. The diversity of signaling pathways led to the identification of numerous biased agonists, such as cariprazine [4,62,63,64]. It should be emphasized that a significant number of DAR agonists for the treatment of PD target D3R more selectively than D2R [63]. However, caution should be exercised in any extrapolation between the animal models of PD and humans regarding the consequences of DA neuron degeneration on plastic changes in DARs in response to chronic DA depletion. The striatum, where the DARs are expressed at a high level, receives a major proportion of the DA projections from the SNc. The cells of the ventral tier of the SNc that project to the dorsolateral part of the striatum appear to be the most vulnerable. Moreover, the depletion of DA is particularly pronounced in the dorsolateral putamen [65,66], a brain area where DARs are considered to be more susceptible to change, while the DA neurons of the mesocortical and mesolimbic systems are relatively unaffected; consequently, the loss of DA in other brain regions has been found to be of less magnitude. The DA innervation of the prefrontal cortex is low compared to the striatum, and the impact of DA degeneration in PD on this brain area is still unclear.

The profound heterogeneity of the loss of DA fibers across the brain may participate in the heterologous changes reported on DAR densities and/or coupling efficacy in different parts of the brain. This could be dependent on the cell type expressing the receptor, the addressing of the receptor in the cell, and the inherent coupling to a specific signaling pathway [4]. Various studies carried out in animal models as well as in patients with PD have reported alterations regarding the density of D1Rs and D2Rs. Indeed, in the model using SH-SY5Y cells stably expressing D1R, the expression of LRRK2 G2019S mutant has been shown to strongly alter D1R internalization upon dopamine treatment, presumably as a result of altered endocytosis [67]. Transgene expression causes an increase in D1Rs in the plasma membrane fraction that correlates with a decrease in the vesicular fraction [68]. There is a large body of experimental evidence suggesting that inflammatory processes are involved in neuronal cell death in PD. It has been shown that the D1R contributes to the control of the immune system through the negative regulation of the inflammasome [69], and that LRRK2 appears to play an important role in inflammatory cells [67]. In addition, the mutant form of LRRK2 (mutations in residue R1441) induces impaired D2R-mediated functions, which potentially could be a pathogenic precondition for DA neuron degeneration in PD [70].

The results from studies using human induced pluripotent stem cell (iPSC) technology have shown that the G2019S mutation of LRRK2 in DA neurons has a significant and direct impact on the membrane localization of D3R, a process which could contribute to the vulnerability of this neuronal population. Interestingly, these molecular alterations seem to be reversible, which opens a new avenue of research in LRRK2 kinase inhibitors for the treatment of PD [70].

There is increasing evidence for a substantial role of the D3R in normal locomotor behavior. Since its expression and distribution profile is significantly modified in specific brains regions, the D3R may have significant implications in PD.

GP is the main target for PD therapy, since it is the principal striatal output brain structure to the thalamus that regulates involuntary movements. Furthermore, high levels of D3R—but no D3R mRNA were found in a primate model of PD, indicating that the D3R is expressed by incoming afferents [71]. In humans, D3R mRNA is located in the striatal medium spiny neurons that project to the internal segment of the GP [59]; therefore, D3R upregulation may appear in this neuronal group at the terminal (GPi) as well as at the somatodendritic (putamen) levels [2]. Such D3R upregulation could induce excessive inhibition of GP targets and a significant decrease in GP firing, which have been causally associated with l-3,4-dihydroxyphenylalanine (l-DOPA)-induced dyskinesia (LID) in 1-methyl-4-phenyl-1,2,5,6-tetrahydropyridine (MPTP)-treated monkeys and parkinsonian patients [72], leading to thalamic nuclei disinhibition and culminating in the overactivation of cortical motor regions. However, a distinction of functionally separate GP divisions must clearly be stated.

In rodents, D3Rs decline in the nucleus accumbens and striatum as a result of DA depletion [73,74,75,76]. It should be noted that in the MPTP-lesioned rat model of PD, receptor binding experiments have revealed a reduction in D3R receptor affinity and D3R mRNA in the nucleus accumbens, although the ventral striatum is affected to a lesser extent [77]. Lower levels of DR3 were also found in the striatum of 6-hydroxydopamine (6-OHDA, a DA toxin) or MPTP-treated monkeys [2,71,77]. In comparison, there is no change in D3R expression in the post-mortem tissues of parkinsonian patients [78]. These discrepancies in D3R expression could be attributed to disease progression and the impact of l-DOPA therapy. It is interesting to note that D3R expression is mostly absent in the normal non-DA-depleted dorsal striatum in rodents and is predominantly low in primates [75]. Since the limbic striatum seems to be involved in neural functions, including locomotion and movement, mesolimbic DR3 could play a significant role in relieving PD symptoms [79]. In 6-OHDA-lesioned rats, D3R-preferring agonists, used at doses that have an inhibitory effect on normo-sensitive rats, induced locomotor stimulation, suggesting a benefit of D3R-preferring agonists as an anti-parkinsonian treatment in DA-depleted animals [80].

Based on the post-mortem studies of PD cases, a decrease in the density of D3Rs by 45% in the BG and an increase in D2Rs by 15–25% in similar brain areas has been shown [81]. In addition, a clear correlation between non-responders to anti-parkinsonian drugs and the lowest level of D3Rs number has also been observed. In contrast, another study showed a correlation between the responses to anti-parkinsonian drugs and elevated levels of D3Rs [82]. Interestingly, the early stages of PD are marked by a decrease in D3R expression; however, the administration of l-DOPA causes an increase in D3R numbers. Prolonged-life DA agonists are intended to alleviate signaling by reducing the receptor numbers. For example, pergolide (known as a D2R agonist) has been reported to reduce D3R levels within both lesioned and intact hemispheres of hemiparkinsonian ratsv [83]. In contrast, the initiation of treatment with l-DOPA, the most used and effective therapy for PD, leads to an increase in D3R numbers, reflected by increased D3R-binding sites and mRNA coding for D3Rs in dynorphin-positive striatal neurons, which send projections to the SNr where D3R is normally expressed at moderate levels [74]. It should be pointed out that l-DOPA does not arrest the progression of the disease. The additional loss of mesolimbic DA neurons results in a continued reduction in D3R numbers in critical striatal areas [79]. In patients with advanced PD, the antiparkinsonian drugs become less effective at treating parkinsonian symptoms, and the patient is considered to be unresponsive [79]. Long-term l-DOPA treatment is responsible for invalidating abnormal involuntary movements, known as LID, which constitutes a serious limitation in the use of this therapy.

## 5. The Repercussions of DA Depletion on the Responsiveness of the Dopaminergic Signaling Pathways

The DA-depleted striatum produces exaggerated molecular responses in numerous signaling pathways in response to DAR stimulation, which may reflect the hypersensitivity of DARs. In the context of DA depletion, the D1R hypersensitivity due to the lack of D1R activity is manifested in exaggerated responses to acute DAergic effects in various signaling pathways. However, there have been no significant changes in the basal levels of expression, illustrated with the expression of dynorphin, substance P, and the D3R dependent of D1R tonic regulation, the maintenance of which requires periodic D1R stimulation [84].

**Figure 2:**
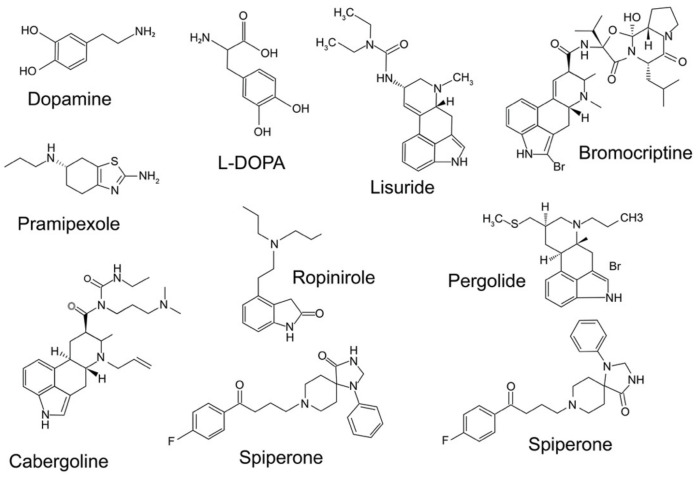
Structure of some important pharmacological agents for dopamine receptors, including dopamine and l-DOPA.

The first description of this hypersensitivity was carried out in rats unilaterally lesioned with 6-OHDA. In these conditions, the animal exhibited rotations when exposed to DAergic drugs at doses that were 10 to 100-fold lower than those expected to induce significant behavioral effects in non-treated animals. In this animal model, rats treated with direct DA agonists, such as apomorphine, (Figure 2) elicit contralateral rotations toward the injured side, where the DARs are supersensitive. Conversely indirect agonists, such as amphetamine, promote the efflux of DA from the intact side and induce ipsilateral rotations to the lesioned side. These rotations reflect the imbalance between the lesioned and the intact hemispheres. The main exception in this schema is l-DOPA, which induces net contralateral rotations as a DAR agonist, while having been assumed to “restore” DA in the denervated side. Beyond the role of serotonergic neurons in releasing DA derived from exogenous l-DOPA, it is acknowledged that l-DOPA does not simply work as a DA precursor [16,85].

The hypersensitivity of some DARs appears to be a consequence that reflects a process of adaptation to the loss of DA stimulation aiming to counteract the reduced availability of endogenous DA. However, the molecular signaling mechanisms remain to be determined.

Both behavioral and electrophysiological experiments have shown a substantial increase in D3R activity, thus exhibiting a hypersensitivity status comparable to that of the D1R [86,87]. D3R supersensitization could be related in part to the interaction of D3R with its truncated splice variant, D3Rnf. D3Rnf could thus impair the responsiveness of the D3R to ligand coupling. Therefore, hypo- and hyperdopaminergic conditions may modulate the D3R/D3Rnf ratio [88]. In the denervated state of DA, D3Rnf may affect the regulatory pathways of the D3Rs and result in D3R hypersensitivity [88]. In addition, changes in the D3R/D3Rnf ratio have been reported in other pathologies involving DA, such as schizophrenia [89] and nicotine sensitization [90], highlighting the potential contribution of the D3Rnf isoform to the homoeostasis of D3R functionality in pathologies associated with defects in DA transmission. The co-expression of D3R and D3Rnf appears to result in fewer binding sites for the D3R compared to the expression of the D3R alone. D3Rnf, presumably due to its homology to the D3R, could integrate into the D3R network and thus interfere with ligand binding to the D3R. It should be noted that the co-expression of D3Rnf has no effect on D2R binding densities [91]. It was shown that the D3R expressed in Sf9 cells was detected by DA agonists as a single binding site. Furthermore, the agonists did not seem to have an effect on forskolin-stimulated adenylyl cyclase activity, suggesting the absence of the appropriate G protein(s) required for D3R coupling in distinction to the D1R responses [92].

At the molecular level, the DA-induced activity of adenylyl cyclase has been reported to be elevated, leading to the accumulation of cAMP, which could be involved in the circling behavior in hemiparkinsonian rats [93,94,95,96,97,98,99]. Moreover, the DA-stimulated accumulation of cAMP has been shown in the striatum of PD patients [97,100]. Downstream pathways and signaling targets of cAMP-dependent phosphorylation are also impacted. In rodents unilaterally lesioned with 6-OHDA, l-DOPA, presumably via acute DA stimulation, induces a higher level of DARPP-32 (DA- and cAMP-regulated phosphoprotein 32 kD) phosphorylation at Thr34, without amending the basal level of DARPP-32 phosphorylation. DARPP-32 is the substrate of protein kinase A (PKA) [101]. The GluR1 subunit of the α-amino-3-hydroxy-5-methyl-4-isoxazole propionate (AMPA) receptor, another PKA substrate, displayed increased levels of phosphorylation at the PKA site, Ser845, induced by acute D2R/D3R stimulation without the modification of the phosphorylation level at the calcium/calmodulin-dependent protein kinase II (CaMKII) site, Ser831. DARPP-32 is considered an important element of DA signaling and is highly expressed in medium spiny neurons. DARPP-32 is converted into an inhibitor of protein phosphatase-1 through its phosphorylation at Thr34 by PKA [102,103].

Interestingly, the development of qualitative changes in D3R signaling may be a contributing factor to the pathophysiology of PD. Indeed, a switch in D3R signaling has been reported in striatal MSNs and striatonigral terminals in the 6-OHDA rat model of PD [86,104]. Thus, physiologically, D3R signaling inhibits CaV1 (L-type) Ca^2+^ channels through protein phosphatase 2B (PP2B) activation. In DA-depleted mice—but not in control animals—D3R modulates CaV2.1 (P/Q-type) via phosphoinositide hydrolysis, leading to the production of inositol trisphosphate (IP3) [86]. Since the liberation of GABA from the striatal terminals is mediated via CaV2.1 channels, D3R signaling through the phosphatidylinositol-4,5-biphosphate (PIP2) pathway may decrease GABA release, thereby affecting synaptic transmission between striatal neurons as a result of DA depletion.

Importantly, the lesion-induced functional hypersensitivity of DARs leads to the activation of various striatal genes as a result of acute DA stimulation. Interestingly, a large number of these genes are related to inducible transcription factors, such as c-Fos, which, in association with other transcription factors, such as the activator protein-1 (AP-1), binds to the AP-1 promoter site and may trigger the expression of other genes. The adaptive mechanisms of striatal neurons to DA neuron degeneration are essential points to be considered in the imbalanced responses to DAR agonists between the denervated and non-denervated sides in the 6-OHDA rat model of PD.

## 6. Molecular Consequences of l-DOPA Administration in the Context of Dopamine Depletion

Although DA replacement therapy with l-DOPA provides an effective treatment for patients with PD, unfortunately, two problems that are posed by long-term administration of this agent remain important issues to resolve: (1) its therapeutic effectiveness is progressively compromised as the disease advances and (2) patients exhibit motor fluctuations and develop severe motor complications.

The problems raised by these adverse effects cannot be resolved with varying appropriate dosing and the administration of l-DOPA and/or DAR agonists. Thus, a detailed understanding of the changes in the signaling pathways induced by DA depletion and by different types of DA substitution treatment may help in designing effective therapies with minimal side effects. The D2R exhibits two distinct variants—the D2SR short and D2LR long isoforms generated through alternative splicing of an 87-bp exon between introns 4 and 5 [105]. The D2SRs are primarily expressed by DA neurons as autoreceptors [106]. Additionally, both of these receptors display either a high-affinity state or a low-affinity state for DA, whereby the D2R high affinity state would be the functional state in the nigral neurons’ DA terminals (presynaptic receptors) [107]. While this last point has not been conclusively documented, results from studies using brain slices have shown that 90% of D2Rs are in the high affinity state. The high affinity state of D2Rs has the potential to be rapidly switched to the low affinity state by the guanine nucleotide [108].

Pharmacologically, a distinctive feature attributed to the D3R, compared to the D2R, is that the D3R is not changed into its low-affinity state by GTP. Thus, D3R has a higher affinity for DA than the D2R. Furthermore, any structural or functional alteration, whether modest or not, strongly affects synaptic signaling, suggesting a crucial role for D3Rs in DA transmission [104,109].

It should be noted that DA depletion exacerbates the indirect pathway (Figure 1) to the detriment of the striatal circuitry, leading to the hyperactivity of the striatal output nuclei, thereby intensifying the net inhibitory striatal output to the thalamus [6]. It is believed that the better DA tuning on striatal circuits offered by DA therapies, notably on the direct versus the indirect pathway, could explain the reduced activity of GPi neurons [110,111]. However, the reduction in activity of GPi neurons induced by long term l-DOPA treatment measured in the thalamus can occur without the enhancement of striatal DA release [112]. In the absence of an appropriate striatal inhibitory control, the motor cortex is uninhibited, leading to the overactivity of the striatonigral pathway providing a direct GABAergic connection, through which the striatum inhibits the output areas of the BG, the SNr, and the GPi, leading to dyskinetic movements [113,114]. The differentiation between the parkinsonian and dyskinetic states cannot be summed up as just opposition between high (parkinsonian state) and low (dyskinetic state) output of the striatal circuit. Indeed, several complex impairments of activity patterns in the striatal system and beyond could be involved.

D3Rs are thought to be implicated in the control of basal DA levels [115]. D3R deficiency has been shown to result in reduced DA tonus [115,116]. l-DOPA may act as a non-selective agonist principally on the most widely expressed receptors: striatal D1R, D2R, and D3R. The therapeutic effect of antiparkinsonian drugs is achieved through selective D1R as well as D2R/D3R agonists in humans and animals [117,118,119,120,121,122].

In the striatum, D3R displays changes in both its expression and function. It has been reported that its ectopic expression in the striatum develops in response to l-DOPA application, and it is thought to be an active component in behavioral sensitization [69].

l-DOPA reverses the upregulation of D2R numbers caused by the loss of DA not only in human PD patients but also in animal models of PD [72,83,123,124,125,126,127]. l-DOPA therapy in mice presenting a complete loss of DA neurotransmissions induces locomotor activity that is significantly higher than that of treated WT mice, as well as an extracellular signal-regulated kinase (ERK) and c-fos responses [128,129]. In addition, l-DOPA reverses the upregulation of Gαolf in hemiparkinsonian rats [94]. Furthermore, the hypersensitivity of the ERK pathway was found to be reversed by l-DOPA, both in MPTP-lesioned monkeys and 6-OHDA-lesioned rats [83,130]. A similar reducing effect of l-DOPA has been reported with the exacerbation of basal CaMKII phosphorylation [131,132] and DARPP-32 at Thr75 [131]. Moreover, chronic l-DOPA administration is successful in reversing, or at least meaningfully improving, the effect of DA depletion in the expression of the super-responsiveness of several immediate early genes (IEGs; c-fos, junB, junD, and c-Jun) [133,134,135]. However, the role of signaling mechanisms induced by the striatal loss of DA in the motor deficits and the reversal of these modifications by l-DOPA in its therapeutic activity remains to be clarified. The intrastriatal administration of a selective CaMKII inhibitor maintains the motor functions in 6-OHDA-lesioned rats, suggesting a link between the hyperphosphorylation of CaMKII and the lesion-induced motor dysfunctions [132].

In hemiparkinsonian rats and MPTP-lesioned monkeys, l-DOPA does not reverse the upregulation of enkephalin [83,84,136,137,138,139]; the enhancement of enkephalin expression was even noted in animal models of PD (rats and monkey) and human dyskinetic PD patients [136,138,140]. l-DOPA is unsuccessful in reversing the effect of DA depletion on arrestin and GRK expression in 6-OHDA-lesioned rats. This is also the case for pergolide (a long-lived DA agonist) (Figure 2) [141]. However, l-DOPA has proven to be successful in reversing the lesion-induced upregulation of arrestin2 and GRK6 [130]. It is noteworthy that pergolide and other D2R/D3R agonists can also bind other receptors [16]. The loss of DA appears to induce some permanent modifications in signaling mechanisms that once generated are not reversed by DA. Acute l-DOPA treatment induces contralateral rotational behavior in hemiparkinsonian rodents, while chronic l-DOPA administration leads to a gradual increase in the frequency of the rotations [75,76,83,141,142]. Moreover, l-DOPA induces a marked upregulation of the activity of dynorphin-positive striatal neurons projecting to the SNr, a region that moderately expresses D3Rs in hemiparkinsonian rats. However, when l-DOPA is administered chronically, this latter effect appears to be increased [74,75,76,83,84,143]. The lesion-induced hyperphosphorylation of cyclic AMP-response element-binding protein (CREB) seemingly becomes worse with chronic l-DOPA administration; in response to DA challenge [144], lesioned rats exhibited DA stimulation but limited or no variation in basal activity.

In the non-lesioned striatum, DA contributes to the negative regulation of the Akt-GSK3 pathway through D2R and D3R [145,146]. Nevertheless, under DA depletion conditions, DA appears to exert positive regulation on Akt-GSK3 signaling, likely mediated by D1R.

### 6.1. Implication of D3 Receptors in l-DOPA-Induced Dyskinesias

The loss of DA neurons in the nigrostriatal pathway can approach 30% without displaying parkinsonian symptoms, and the motor symptoms begin to be observed when the loss of DA in the putamen reaches 70% [10]. These generated changes could seriously modify the density and/or sensitivity of post-synaptic DAR subtypes, including D3R [147]. The neurons of the direct pathway circuits, which connect the striatum to the output nuclei, project to the GPi and SNr and express D1Rs. Neurons of the indirect pathway, which establish connections with the output structures involving the GPe and STN, express D2Rs [148]. The disinhibition of thalamocortical neurons through the activation of the direct nigrostriatal pathway promotes locomotor activity, while thalamocortical inhibition via the activation of the indirect pallidostriatal pathway decreases locomotor activity [149].

It should be pointed out that the pharmacokinetic of l-DOPA is subject to change due to fluctuations in the effective plasma dose over time, thus inducing a sharp transition between the active treatment phase “on” and the inactive treatment phase “off” [150].

The response to l-DOPA treatment has been shown to be exacerbated following chronic l-DOPA administration but then gradually disappears upon withdrawal of the treatment regimen. The change in rotational behavior has been reported to be paralleled by D3R protein appearance and disappearance in the caudate putamen. In addition, SKF 38393, a selective D1R acting as a partial agonist, produces similar D3R induction as l-DOPA, although it is less extensive. l-DOPA-mediated D3R induction has been shown to be significantly prevented by SCH 23390, a D1R antagonist [75].

The overexpression of D3R was found in dyskinetic monkeys, while downregulation of D3R was caused by MPTP-induced DA lesions and was re-established to its normal level in non-dyskinetic animals [2]. A partial D3R agonist significantly improves the LID without impairing the effect of l-DOPA, likely through a decrease in D3R-mediated signaling, suggesting that the hypersensitivity of DA is not only reversed but intensified by l-DOPA, leading to LID. In addition, the incidence and severity of LID seem to be correlated with D3R density in the putamen. D3R antagonists have shown the ability to mitigate dyskinesia accompanied by the development of PD-like symptoms. LID and PD-like symptoms could presumably be associated with significant D3R down- and upregulation. Accordingly, normalizing D3R function by partial agonists may prevent the side effects of l-DOPA treatment in parkinsonian patients [2].

Pharmacologically, some investigators have reported that D3R antagonism was effective in reducing LID [151,152], while other groups failed to support this result [153]. The fact that D3Rs and D2Rs are found in overlapping regions, and, on the other hand, the lack of selectivity of the compounds used for the D3R over the D2R may be contributing factors to this apparent inconsistency. Moreover, the pathophysiology of LID may imply other DAR subtypes in addition to the D3R. Thus, L-745870, known as a selective D4R antagonist, has been reported to reduce LID in the macaque model of PD. DA and its agonists have also been shown to have a higher affinity for the D3R than for the D2R [1] (Table 1).

Some studies have suggested that the deprivation of the anterogradely transported factor, brain-derived neurotrophic factor (BDNF), normally produced by DA neurons, appears to alter D3R expression not only in animal models (6-OHDA-lesioned rats and MPTP-treated primates) but also in PD patients [74]. The reduction achieved by BDNF deprivation appeared to be selective, since D1R and D2R expression remained practically unchanged [75,183]. In 6-OHDA-lesioned rats, it has been reported that sensitization development has been associated with increasing levels of D3R mRNA and binding sites in the denervated caudate putamen, a brain region normally lacking in this receptor [75]. An increase in BDNF mRNA was also observed with l-DOPA mainly in the deep cortical layer V containing pyramidal cell bodies that project to different subcortical regions, including different accumbal and striatal regions [184], suggesting that l-DOPA may increase the release of striatal BDNF from corticostriatal neurons [183]. The improved behavioral response mediated by l-DOPA may presumably underlie the progressive motor recovery experienced when treatment is initiated, or the development of LID in long-term l-DOPA-treated patients [185]. This is consistent with the correlation between D3R upregulation detected in the GPi and the putamen and the severity of dyskinesia [59].

**Figure 3:**
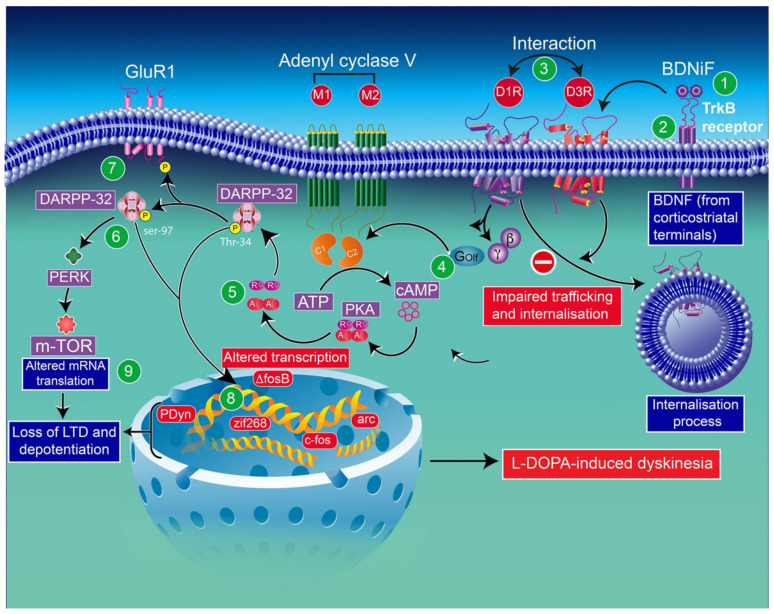
Schematic representation of D3R and D1R interaction through some abnormalities in DAergic signaling caused by sensitized D1R signaling in the context of LID: D3R overexpression in the D1-enriched direct GABA pathway may contribute to the sensitization of D1Rs and the development of LID [70]. The enhancement of BDNF release (1) by chronic treatment with l-DOPA elicits the activation of TrκB (2) receptors and induces the overexpression of D3Rs. The levels of membrane-bound D1Rs increase through direct interaction with D3Rs (3). Thus, exacerbated D1R sensitization has been associated with dyskinetic behavior. D1R-induced increased levels of adenylyl cyclase (mostly the AC5 isoform) (4) through the stimulatory heterotrimeric Golf α subunit results in the production of cAMP, leading to hyperactivation of PKA (5) and DARPP-32 (6). Either pharmacological inhibition of PKA or genetic inactivation of DARPP-32 appears to reduce LID. Thus, abnormalities in PKA/DARPP-32 signaling seem to increase the phosphorylation of the GluR1 subunit of the AMPA receptor (7). The subsequent increase in striatonigral MSN excitability through D1 receptors may be involved in the loss of the expression of corticostriatal long-term depression (LTD) and long-term potentiation (LTP) (8). The activation of ERK through sensitized D1R mediates transcriptional processes. The inhibition of ERK signaling was found to counterbalance the induction of LID. CREB phosphorylation, in response to PKA/DARPP-32 and ERK/MSK1 signaling, im-proves the expression of immediate early genes (ΔfosB) and prodynorphin (8). ERK activation may improve mTORC1 complex-dependent signaling, thereby initiating mRNA translation and protein synthesis (8). mTOR-mammalian target of rapamycin; DARRP-32-dopamine- and cAMP-regulated phosphoprotein of 32 kilodaltons; PERK-RNA-dependent protein kinase (PKR)-like ER kinase; BDNF-brain-derived neurotrophic factor; PKA-protein kinase A; cAMP-cyclic adenosine monophosphate; PDyn-prodynorphin; Zif268-zinc finger family 268.

In line with these results, in l-DOPA-treated hemiparkinsonian rats, administration of a BDNF antagonist into the denervated striatum impedes the induction of both D3R mRNA and protein expression [183]. In addition, the infusion of the BDNF antagonist results in a dose-dependent inhibition of behavioral sensitization to l-DOPA [75]. l-DOPA-induced sensitization in rats [75] and the development of dyskinesia and drug-induced upregulation of D3Rs in monkeys [2] have also been reported. The mechanism underlying this regulation appears to imply the involvement of D3R expression dependent on BDNF [183]. Indeed, in mice lesioned with 6-OHDA, BDNF expression appears closely associated with prolonged l-DOPA treatment. This finding indicates that the activation of D1Rs is involved in modulating BDNF production [183], resulting in an overexpression of BDNF-TrκB receptors, in this animal model, which has been reported to increase the levels of D3Rs in the striatum, leading to D1R-dependent pathway exacerbation, and thereby leading to LID [2,183] (Figure 3).

Striatal Shp-2 has been shown to be involved in both the behavioral and molecular features of LID. Indeed, the availability of Shp-2 to interact with the D1R is believed to be relevant to the development of LID. A striatal biochemical analysis showed that Shp-2 levels were linearly associated with the severity of LID [186].

It is important to note that the alterations in D3R expression, localization, and function induced by chronic l-DOPA administration may be responsible for both beneficial motor effects and motor deficits. Thus, the D3R should be considered to be involved in the pathophysiology of PD, and thereby could prove to be a promising therapeutic target in the treatment of PD.

### 6.2. Role for D3R in the Control of LID

An implication of D3R in the manifestation of dyskinesia, although strongly suggested, remains unclear [151,153]. In the striatum, D3R displays changes in both its expression and function. It has been reported that its ectopic expression in the striatum develops in response to l-DOPA application and is thought to be an active component in behavioral sensitization [75]. In 6-OHDA (rats and mice [76,152,187]) and MPTP (mice [188] and monkeys [71,187,189]) models of PD, increased D3R expression has been noted in dyskinetic animals. D3R expression may be affected by D1R agonism or antagonism, thereby pointing to possible involvement of D1R stimulation in the activation of D3Rs [75,76].

In the MPTP monkey model of PD, the combination of BP 897 (a partial agonist possessing 70-fold selectivity for D3R over D2R [190]) with l-DOPA reduced LID by 66%; however, it did not exhibit a functional effect of the motor recovery obtained with l-DOPA, presumably due to its lack of selectivity in vivo. It might be noted, however, that the selective D3R antagonist S33084 failed to demonstrate antidyskinetic action in monkeys with previously established LID, indicating that D3Rs are not involved in the maintenance of LID [191]. The potential sources for this discrepancy likely depend on the one hand, on differences in the affinity and degree of selectivity for D3R between S33084 and BP897, and on the other hand, on the differences in D3R expression between different animal models, since elevations in striatal D3Rs are not constantly observed [71,192]. Furthermore, other DAR subtypes could also be involved in LID, since L-745870, known as a selective D4R antagonist, has been reported to reduce the severity of dyskinesia in a progressive MPTP-lesioned macaque model of PD [193].

Furthermore, dyskinetic monkeys exposed to the D3R-selective antagonist ST 198 combined with l-DOPA displayed attenuated LID. However, this effect was accompanied by a recurrence of parkinsonian motor symptoms and locomotor dysfunctions, suggesting that ST 198 action at D3R (and possibly at D2R) may be responsible for the deterioration of the l-DOPA benefit on motor symptoms, reflecting the PD-like symptoms [2]. Contradictory results have been reported in previous findings, in which neither D3R inhibition nor invalidation impaired locomotion in normal rodents [178,194,195]. l-DOPA treatment following DA denervation may reveal a different distribution of the functions of the D3R and the D2R [2]. In addition, unilaterally lesioned rats receiving the D3R-selective antagonist PG01037 displayed attenuated LID [196]. Moreover, the application of the D3R-selective agonist PG01042 attenuated abnormal involuntary movements (AIMs) in a dose-dependent fashion, without inducing motor impairment of l-DOPA-treated animals as in the case of PG01037 [197]. WC-10, presented as a D3R weak partial agonist/antagonist, co-administered with l-DOPA resulted in attenuated AIMs. However, some negative impacts on certain aspects of motor activity were observed. On the other hand, when WC-10 was administered following l-DOPA therapy, AIMs significantly and rapidly improved. Furthermore, WC-44, identified as a D3R agonist and D2R partial agonist, could attenuate AIM scores in a dose-dependent manner but induce motor side effects at high doses, which may be related to the blockade of both D3Rs and D2Rs. Thus, it was suggested that the design of D3R ligands acting as selective agonists with higher D2R/D3R affinity ratios may predict better improvements in their therapeutic efficacy for LID [147].

The precise mechanisms underlying the roles of D3R in LID are still being pursued; however, several observations have suggested that they may involve D1R modulation or the direct activation of D3R. Experimentation with D3R knockout animals has reported the reduced development of LID, while striatal D3R upregulation with subsequent l-DOPA therapy or D3R agonist results in the patterns of stereotypic behavior [198]. Furthermore, the potential dyskinesiogenic contribution of the D3R may involve a D1R–D3R interaction [199]. Moreover, the interaction between D1R and D3R may be reflected in a cross-sensitization [200].

## 7. Neuroprotective Effects of D3 Receptor-Preferring Agonists

There is a presumption that the relief of motor dysfunction in PD requires D2R-like stimulation [79,201,202]. Indeed, the activation of D2R and/or D3R is the basis of the anti-parkinsonian effects of DA agonists [79]. Drugs used for the treatment of PD were initially identified as DA agonists with a high affinity for the D2R. It must be emphasized, however, that they also possess other affinities with a marked preference for D3R. Such is the case, for instance, for ropinirole and pramipexole (PPX), which have a greater affinity for D3Rs than D2Rs.

The D3R-preferring agonists PPX and S32504 are the most effective neuroprotective compounds in 6-OHDA-lesioned rats [202] and in MPTP-treated mice [203,204] and primates [205]. PPX appears to exert a neuroprotective effect in vivo in part through its interaction with D3R. In agreement with these findings, studies carried out on a gerbil model of global ischemia showed a decreased loss of DA neurons, but not of CA1 hippocampal pyramidal neurons. This is in accordance with the D3R distribution in such neuronal groups. This protective effect could result from the release of a trophic factor induced by the receptors.

Using imaging studies of nigrostriatal terminals, the D3R-preferring agonists PPX and ropinirole have been shown to reduce the progression of PD [206]. In addition, PPX (1 mg/kg) confers protection to DA neurons in MPTP-lesioned mice [206,207]. Moreover, PPX contributes to substantially reducing the influence of MPTP on TH-positive neurons of the SNpc, and the DA transporter (DAT) of DA cells [82]. It is believed to be protective through the induction of neuroprotective factors, such as Bcl-2, which can prevent apoptosis [208], and a putative 35 kDa DA neurotrophic factor [209]. MPTP-lesioned mice lacking D3Rs showed attenuation of the neuroprotective activity of PPX.

Furthermore, the D3R/D2R agonist PPX has been shown to have neuroprotective properties against the MPTP toxicity, which were significantly attenuated in D3R knockout mice and also by the concomitant administration of A-437203 (a selective D3 antagonist). It should be noted that A-437203 has no effect in mice lacking D3Rs [210]. However, it should also be pointed out that in these studies, the animals were chronically exposed to DA neurotoxins or received a single injection to cause an irreversible loss of neurons while they were administered the D3R-preferring agonists before the neurotoxin, compromising the pertinence of these results to PD in humans. In addition, a study using DAR agonists with a sub-chronic MPTP treatment protocol failed to detect a significant effect of post-MPTP treatment with PPX in decreasing the toxic effects of MPTP. However, combined pre- and post-treatment with D3R-preferring agonists was more efficient to mitigate its neurotoxic effects than pre-treatment alone [211].

In mechanistic terms, the D3R agonist PPX may provide neuroprotective effects through a regulatory influence on paraplegin (a mitochondrial regulation protein) [212], which is attenuated by GR103691, a D3R antagonist [213], while it is not affected by L-741,626, a D2R-selective antagonist. Moreover, pre-treatment with the newly developed D2R/D3R agonist D-512 improved MPTP-mediated effects on motor response alterations in experimental parkinsonism, indicating its potent neuroprotective role in motor functions against the neurotoxin [214]. Thus, initiating therapy at an early stage with PPX may potentially offer some level of neuroprotective effect. d-264 [(–)-*N*6-(2-(4-(biphenyl-4-yl) piperazin-1-yl)ethyl)-*N*6-propyl-4,5,6,7-tetrahydrobenzo[d]thiazole-2,6-diamine], displaying a preferential agonist activity for D3Rs [215,216], appears to exert its neuroprotective effects by reversing the lactacystin-induced inhibition of proteasome activity. Neurochemical lesioning studies have suggested that d-264 may prevent, at least partially, the loss of neuronal cell populations, potentially due to the inhibition of microglial activation. Additionally, both lactacystin- and MPTP-treated mice exhibited increased BDNF and GDNF levels by pretreatment with d-264. BDNF and GDNF protein upregulation has been proposed to underlie, at least in part, the mechanism mediating the neuroprotective effects of d-264. It is assumed that neurotrophic factors may impair pro-apoptotic signaling pathways. However, the exact molecular mechanisms responsible for the neuroprotective effects of neurotrophic factors remain to be determined. Although lisuride is recognized to be a D2R-like receptor agonist, it has a higher affinity for D3Rs than for D2Rs [157,166,217]. Lisuride was shown to be effective in mitigating the 6-OHDA-induced loss of TH-positive cells when introduced directly into the SN, and also in primary neuronal cultures. L-741,626, a D2R antagonist, failed to attenuate lisuride’s neuroprotective action, whereas GR103691, D3R selective antagonist, was capable of suppressing its neuroprotective effect [218]. The neuroprotective activity of the D3R agonist rotigotine against glutamate toxicity is mediated via the activation of Akt, leading to the abolition of the pro-apoptotic factor glycogen synthesis kinase-3-beta [219]. Aripiprazole, a D2R/D3R ligand with very weak partial agonist properties, also exhibits a neuroprotective activity against glutamate, which is not abolished by a D2R-like receptor antagonist [220].

However, the underlying molecular mechanisms mediating the effects of D3R activation remain poorly understood and there is still no convincing mechanistic model for this effect. Moreover, the pharmacological profiles of lisuride and aripiprazole go largely beyond DAR, and both compounds exhibit tremendously high affinities towards serotonergic receptors [64].

It cannot be excluded that some of the observed effects are related to the free radical scavenging properties of D3R agonists that could confer neuroprotection. PPX reduced l-DOPA-induced cytotoxicity in cultures of primary dopaminergic neurons, and this effect was not blocked by D2R/D3R antagonists [221]. Such an effect could be related to the inactive stereoisomer of PPX (S[+]), which has no affinity for DARs. In addition, PPX has been shown to exert a neuroprotective effect in structures where DARs are lacking (lower olivary neurons) [222]. PPX also exerts a protective effect on human ARPE-19 cells against the toxic effects of H2O2, suggesting an antioxidant effect of pramipexole [223].

## 8. Desensitization Process of Dopamine D3 Receptors

Upon exposure to certain agonists, D3Rs may undergo an agonist-induced decrease in receptor responsiveness over time, known as desensitization [224,225]. Several studies have shown that functional desensitization of D2R-like receptors (D2R, D3R, D4R) occurs to a lesser degree over time than that of D1R-like receptors and necessitate extended agonist therapy [226,227]. Unlike D2Rs, the D3Rs exhibited moderate internalization following DA stimulation. The desensitization and resensitization of the D3R process may occur rapidly and could represent fast feedback on short-term variations in DA levels [228].

Data from hemiparkinsonian rats have shown that the detected lesion-induced downregulation of GRK isoforms in the caudal striatum are reversed by pergolide. l-DOPA, unlike pergolide, is not effective in reversing the downregulation of GRK isoforms caused by DA depletion in the caudal striatum [65]. The GRK-mediated desensitization mechanism operates by lowering signaling and reducing the number of receptors [83], consistent with a pergolide-induced reduction in the concentration of D3Rs in lesioned, as well as the intact hemisphere, of hemiparkinsonian rats [77,83], while l-DOPA appears to upregulate them [83]. This pergolide-related effect may reflect drug-induced D3R desensitization. While the desensitization of D1Rs develops readily through arrestin- and GRK-dependent mechanisms [229], D3Rs are relatively resistant to homologous desensitization [230,231], which might explain why rodents treated with long-lived agonists that selectively target D1R quickly develop tolerance, thereby losing much of the potential effectiveness. This leads to the need to escalate doses without succeeding in restoring the response. Furthermore, the limited availability of GRKs in the motor areas of the striatum could result in the development of LID, or at least instill an environment conducive to LID.

Furthermore, the increased expression of D3R associated with selective desensitization may contribute to the development of motor impairments and LID [224]. Simms et al. recently showed that the performances of hemiparkinsonian rats were improved by treatment with SK609, a D3R agonist having an atypical signaling profile. Additionally, SK609 co-administered with l-DOPA reduces motor alterations caused by l-DOPA [232]. Buspirone, exhibiting a D3R antagonist activity, will be introduced in a clinical trial for LID (NCT02589340). However, its ability to provide therapeutic benefits in LID is associated with its 5-HT1A receptor agonist activity [233].

Interestedly, Westrich et al. suggested that the D3R switches to a different conformational state when subjected to tolerance and proposed that this conformational transition may involve the decoupling of the D3R from the signaling pathways [226].

## 9. D3-D1 Receptors Interactions

### 9.1. Neurobiology of Dopamine D3-D1 Receptors Interactions

The coexistence of D3Rs and D1Rs in the direct pathway indicates a potential interaction between the two-receptor subtypes and could prove beneficial in PD therapeutic strategy. D1R and D3R are co-expressed in the most GABAergic neurons of the striatum [234]. This is supported by a significant induction of mRNA synthesis coding for D3R through D1R activation [76] that is mitigated by D1R antagonist co-administration, and the synergistic interaction between D1R and D3R to modulate substance P expression [234]. The D3R and D1R display cooperativity at striatonigral neurons. Moreover, this cooperative functional interaction has been demonstrated in the striatum [235,236] and on the nerve terminals of the SN [237]. The D3R induces changes in the relative affinity of the D1R. Thus, the increased affinity of D1Rs for DA directly increases cAMP levels and GABA release in striatonigral terminals, which seems to mediate LID when released at high levels.

Depending on the brain areas considered, D3Rs and D1Rs seem to interact in two different ways: (1) in monomeric form (“typical signalization”) in the Calleja islands, where D3Rs seem to antagonize D1R-mediated responses, and (2) in dimeric form (“atypical signaling”) in the dorsal striatum and nucleus accumbens and their projections to the SNr, where the D3R appears to potentiate D1R-mediated responses [235,236,238]. Interestingly, following DA denervation, D3R switches from atypical to typical signaling, particularly in the dorsal striatum, which results in the antagonism of D1R-mediated cAMP accumulation and GABA release. This could likely be due to the loss of the dimeric interaction of D3R and D1R as a result of denervation.

**Figure 4:**
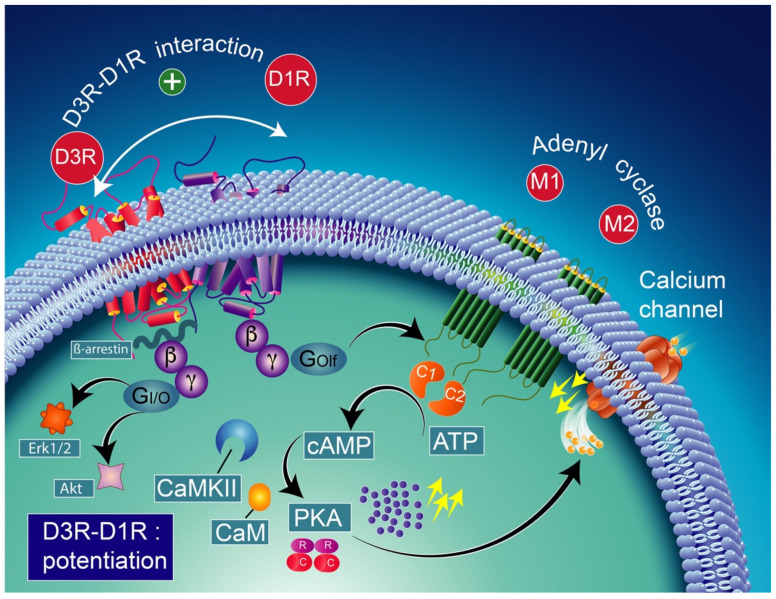
D1R and D3R interaction in basal conditions considering the colocalization of D1R and D3R in medium spiny GABAergic/substance P-positive neurons [248,249]. The activation of the D3R has a synergistic effect on the D1R, increasing the levels of adenylyl cyclase (mostly the AC5 isoform) through the stimulatory heterodimeric Golf α subunit, leading to the production of cAMP [238,250] which results in the activation of PKA and GABA release. CAM-calmodulin; CaMKII-calcium/calmodulin-dependent protein kinase II; PKA-protein kinase A; PDE-cAMP phosphodiesterase; AMP-5′ adenosine monophosphate-activated protein kinase; cAMP-cyclic adenosine monophosphate; Erk1/2-extracellular signal-regulated protein kinases 1 and 2; Akt-ser-ine/threonine kinase.

**Figure 5:**
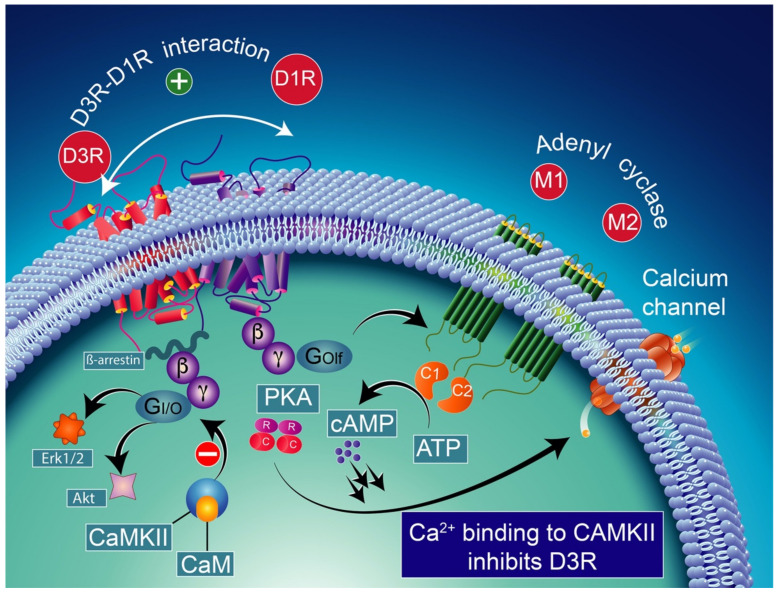
D1R and D3R interaction in high-level Ca^2+^ conditions. Relatively low levels of CaMKII activity induced by low cytoplasmic Ca^2+^ result in the full expression of D3R function through sensitization of D1Rs, which in turn can respond to lower levels of their ligand [237]. The D3R-mediated sensitization mechanism is turned off by activated CaMKII via increased intraterminal Ca^2+^. CAM-calmodulin; CaMKII-calcium/calmodulin-dependent protein kinase II; PKA-protein kinase A; Erk1/2-extracellular signal-regulated protein kinases 1 and 2; Akt-serine/threonine kinase.

**Figure 6:**
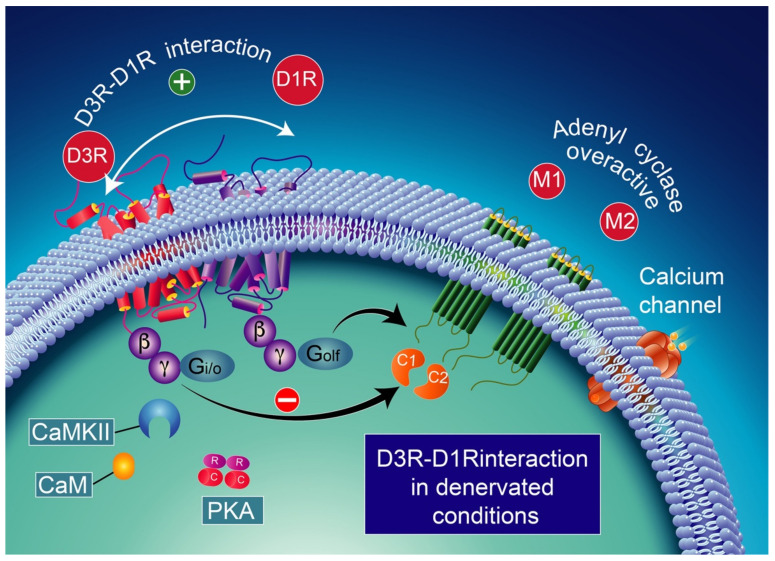
D1R and D3R interaction in denervated conditions. D3R activation switches to inhibitory action upon D1R stimulation. CaMKII activation is no longer expected to occur [246] probably due to the low expression of calmodulin. The D3R activation in denervated dyskinesia rats prevents D1R-induced GABA release at striatonigral terminals. This effect appears to be mediated by an antagonist interaction between the two receptors. CaMKII-calcium/calmodulin-dependent protein kinase II; CaM-calcium/calmodulin.

Note that the concomitant activation of D1R and D3R within the D1R–D3R heteromer leads to G protein-independent and ß-arrestin-mediated activation of Erk1/2 and Akt in both the nucleus accumbens and transfected cells [239]. D3R also promotes the activation of the Akt pathway mediated by phosphoinositide 3-kinase (PI-3K) [240,241], resulting in the activation of the mammalian target of rapamycin (mTOR), one of the major signaling pathways involved in neuronal survival and structural plasticity [242,243]. Importantly, D3R signaling pathways can be modulated by cytoplasmic [Ca^2+^], which involves calcium/calmodulin-dependent protein kinase II (CaMKII) [244,245]. Indeed, D1R signaling is dynamically modulated by the activation of D3R depending on cytoplasmic Ca^2+^ levels that trigger CaMKII, which subsequently phosphorylates the third intracellular loop of the D3R. This phosphorylation interferes with the capacity of the D3R to potentiate D1R signaling [238,244]. Furthermore, in 6-OHDA-lesioned wild-type rats, D3R has been shown to actively abolish D1R signaling in response to D1R stimulation, independent of CaMKII status [246]. Indeed, relatively low levels of CaMKII activity induced by low cytoplasmic Ca^2+^ results in the full expression of D3R function through the sensitization of D1Rs, which in turn can respond to lower levels of their ligand [237] (Figure 4). Thus, the D3R-mediated sensitization mechanism is turned off by activated CaMKII via increased intraterminal Ca^2+^ (Figure 5) [244,245,247]. In denervated tissue, D3R activation has an inhibitory action on D1R stimulation, resulting in a low calmodulin expression and depressed CaMKII response, which is no longer activated by depolarization [246] (Figure 6).

### 9.2. Neurobiology of Functional Heteromeric Complexes of D1-D3 Receptors

Different DAR subtypes are capable of forming a heteromeric complex with D3Rs, generating receptor heterodimers as is the case with the D1R [235,236] and D2R [251]. Thus, four different D3R oligomers have been described: D1R–D3R, D2R–D3R, D3R–D3R, and the A2AR–D3R heteromers. Other D3R-containing heterodimers include heterodimers associating D3R with melatonin (MT) MT1R or MT2R, ion channel receptors, or the nicotinic receptor [252]. According to both pharmacology and functional studies, other types of dimerizations have been suggested, such as with the neurotensin receptor NTS2 [253] and the endothelin receptor ETB [254], although the existence of these heterodimers has not been conclusively demonstrated (see reference [252]).

At the molecular level, filamin A (FLNA) has been found to be involved in the intracellular trafficking regulation of D2Rs and signaling by D3Rs by maintaining the cellular activity of RalA at low levels. Indeed, decreasing the cellular FLNA level results in increased activity of RalA, interfering with both normal intracellular trafficking and D3R and D2R signaling through β-arrestin and GRK2, respectively. Ral, a part of the small GTPase of the Ras family, is involved in receptor endocytosis [255]. Furthermore, GRK2 and β-arrestins were found to be implicated in the blocking activities of RalA acting on D3R-mediated signaling and D2R intracellular trafficking.

**Figure 7:**
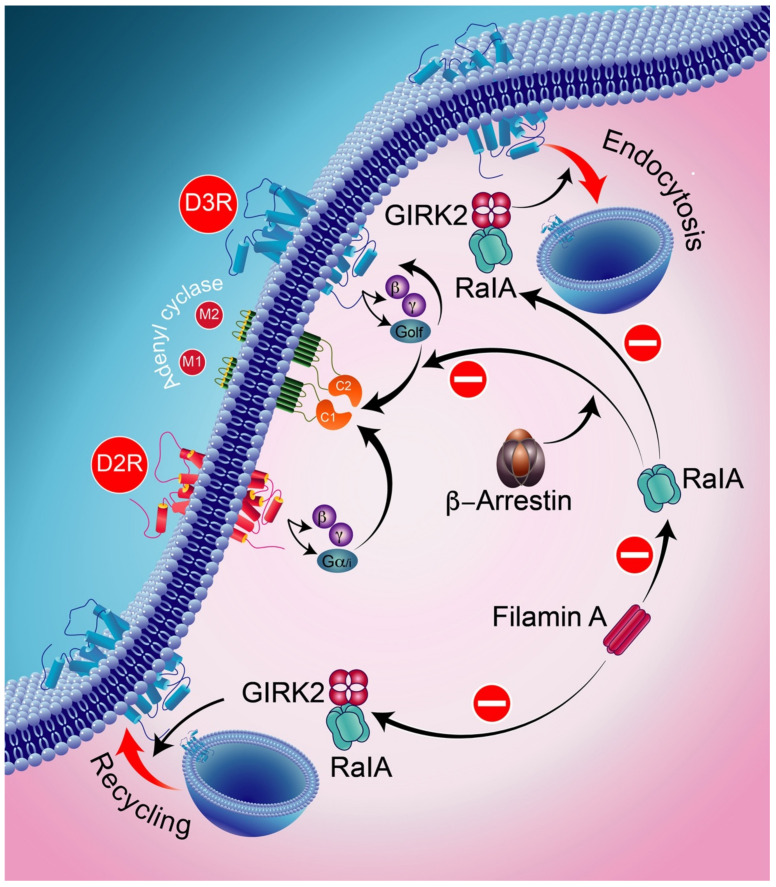
Ral, a part of the small GTPase of the Ras family, is involved in receptor endocytosis. GRK2 and β-arrestins are implicated in the blocking activities of RalA acting on D3R-mediated signaling and D2R intracellular trafficking. RalA is involved in GRK2-mediated receptor phosphorylation in the constitutive recycling and resensitization of D2Rs. The D3R, through its constitutive action with β-arrestin, binds constitutively to active RalA, thereby resulting in the constitutive inhibition of D3R signaling. GIRK2-protein-regulated inward-rectifier potassium channel 2.

In addition, RalA is involved in GRK2-mediated receptor phosphorylation in the constitutive recycling and resensitization of D2Rs [256]. The D3R, through its constitutive action with β-arrestin, appears to bind constitutively to active RalA, thereby resulting in the constitutive inhibition of D3R signaling (Figure 7). In the following, we will summarize the major findings related to the D1R–D3R complex.

There is evidence indicating that abnormal D1R signaling plays a major role in the development of LID, notably through the overstimulation of the cAMP/PKA/Erk1/2 pathway due to the increased coupling of D1Rs to G proteins [101,257,258]. Though a large number of animal models have revealed impaired D1R function associated with LID, including MPTP-lesioned monkeys [239,259] and 6-OHDA-lesioned rodent neurons [101,257,258]), an increasing amount of evidence indicates that D3R signaling may contribute significantly to the molecular processes underlying dyskinesia [75,76].

The potential role of D3R in dyskinesia has been supported by a variety of studies. Indeed, upregulated D3Rs induced by haloperidol in nonhuman primates’ striatum were associated with tardive dyskinesia [240]. Furthermore, the viral overexpression of D3R in the striatum was found to induce dyskinetic movements [260]. Moreover, while genetically engineered mice have shown that deletion of the D3R reduced LID without affecting the antiparkinsonian benefits of l-DOPA [152], the administration of a D3R antisense oligonucleotide prevents l-DOPA-induced behavioral sensitization in 6-OHDA-lesioned rats [152,261]. The l-DOPA-induced elevation of D3R expression was higher in D1R than D2R-containing striatal medium-sized spiny neurons, suggesting that D1R and D3R may act synergistically to modulate LID [152].

In support of this view, the role of D3R in LID has been identified through the use of the antisense-induced suppression of D3R expression in the striatum [261]. Furthermore, the importance of D3R for dyskinesia is highlighted by the overexpression of D3R in the dorsal striatum, which was found to intensify dyskinesia states [260]. Since the D1R–D3R heteromer was detected in the striatum, the intensification of D1R signaling through the enhancement of D3Rs in D1R-expressing MSNs may provide a mechanism by which impaired D1R–D3R function could contribute to the development of LID.

Thus, the D1R–D3R heteromer has been hypothesized to play a key role in the pathophysiology of neurological disorders, such as drug addiction and LID [198,262]. Indeed, the D1R is critical in the development of LID [263,264]. Furthermore, considering both that D1R-mediated signaling in the striatum [218,219] may be potentiated by D3R activation and D1R internalization may be regulated by D3R [265], the D3R could also play a significant role in the pathophysiology of LID. However, this must be understood within the context of an upregulation of D3R [198,266]. Since the D1R–D3R heteromer may facilitate the stimulatory coupling of the D1R to adenyl cyclase, one could consider that the D1R–D3R heteromer may represent a pharmacological target that elicits a different pharmacodynamic effect depending on the allosteric interactions within the D1R–D3R complex. 

The overexpression of D3R in the D1R-enriched direct GABA pathway in the context of DA denervation and intermittent l-DOPA therapy seems to contribute to causing D1R sensitization and the development of l-DOPA-induced dyskinesias [76,267]. Thus, facilitating the interaction between D3R and D1R could be a promising therapeutic target for the treatment of PD. It is therefore of fundamental interest to identify D1R/D3R heteromers at the cellular level and demonstrate D3R/D1R receptor interactions in striatal cells [235,236]. In line with these findings, in reserpinized mice, D3R activation improves the locomotor-stimulating effects of D1R agonists. These findings support the view that the D1R–D3R heteromer may act by improving the recognition and signaling of D1Rs, particularly concerning D1R-mediated locomotor activity, and when heightened by chronic l-DOPA therapy, may also contribute to the onset of dyskinesias in parkinsonian patients [236].

### 9.3. Pharmacological Significance of D3–D1 Receptor Interaction

In the advanced stages of PD, several major symptoms of the disease are no longer rescued by drug therapy. At this point, the patient is considered as unresponsive. Chronic l-DOPA administration in monkeys rendered parkinsonian with MPTP led to LID symptoms in 55% of cases [2]. The DA denervation and intermittent l-DOPA therapy result in D3R overexpression in the D1R-enriched direct GABA pathway, which could contribute to the sensitization of D1Rs and the development of LID [76]. Two observations have suggested that plasma membrane D1R expression is upregulated in animal models of LID [265,268]. Interestingly, in dyskinetic animals, the D1R preserves its ability to internalize following D1R agonist stimulation [265], suggesting that the D1R remains actively anchored at the plasma membrane when linked to a ligand. Furthermore, D1R is co-expressed with D3R receptors in the striatonigral MSNs [76]. In addition, the dorsal motor-related striatum showed elevated levels of the expression of the D3R in experimental rats [75], mice [269], and monkey [2] models of dyskinesia.

The D1R has been reported to directly interact with the D3R [235] via intramembrane D1R–D3R crosstalk [236]. The disturbance of such crosstalk may contribute to LID. In agreement with this proposal, the administration of ST 198 (a specific D3R antagonist) [2] in 6-OHDA-lesioned animals altered the subcellular distribution of D1Rs in dyskinetic rats. Furthermore, it has been shown to reduce the severity of dyskinesia in both rodents and non-human primates [2,151]. Furthermore, when co-administered with l-DOPA, it reduces AIMs, which is consistent with the decrease in dyskinesia observed at the same time with D3R antagonists [265]. This is consistent with the synergistic interactions between D3R and D1R [151,153,196]. Therefore, the functional interaction between the D1R and D3R receptors may be critical for LID development, presumably through a possible link between their activation states and crossing effects mediated by the two receptor subtypes [75].

In D1R-expressing MSNs, the increased expression [2,75,76,270] and ectopic localization of D3R have been reported in the striatum of 6-OHDA-lesioned rats following chronic treatment with l-DOPA. This D3R ectopic overexpression [2,75,183,265,270,271] could facilitate the activation of the D1R direct pathway through D1R–D3R interactions [265,271], since D1R–D3R heteromers are reported to be functionally active [236,249]. D1R activation promotes adenylyl cyclase signaling, thereby resulting in increased cAMP levels and consequently the PKA-catalyzed phosphorylation of DARPP-32 at threonine 34, which converts it into an inhibitor of protein phosphatase-1 (PP1) [102,103]. Furthermore, the simultaneous injection of D1R and D3R agonists was found to synergistically intensify dyskinesia. Thus, the hyperphosphorylation of protein kinase A, ERK1/2, and DARRP-32 in the striatum has been associated with LID [258,272,273]. Furthermore, it was assumed that pERK1/2 expression was widely mediated by the D1R since this effect is suppressed by D1R antagonists. It was reported that the combination of D1R and D3R agonists resulted in the synergistic induction of pERK1/2, which could represent a biochemical “hallmark” of the D1R–D3R heteromer [274].

## 10. D3 Receptors, l-DOPA, and DA

As mentioned at the beginning of this review, l-DOPA is part of the field of DAR agonists. l-DOPA should have induced ipsilateral rotations in unilaterally 6-OHDA-denervated rats similar to amphetamine if it was acting as a DA precursor in spared DA terminals, and not contralateral rotations as the agonists at DARs. While it has been indicated that the contralateral rotations in this model are predictive of an antiparkinsonian activity of selected drugs, it surely cautions the tentative hypotheses explaining its efficacy via the involvement of remaining DA terminals. The latter hypothesis, still conveyed by several authors, is even more difficult to support when taking into account that l-DOPA inhibits the electrical activity of DA neurons [275], even in partially DA-denervated rats [276]. The rotation of l-DOPA in the 6-OHDA rat model of PD is uncoupled to its action on striatal DA release, in variance with the rotations induced by amphetamine [277]. The increase in extracellular DA levels induced by l-DOPA does not mobilize spared DA terminals and instead involves serotonergic terminals [278,279,280]. Because striatal serotonergic terminals are much less represented than the physiological density of striatal DA terminals, the level of extracellular DA induced by l-DOPA is much lower than the physiological levels of striatal DA [16]. l-DOPA promotes a hypodopaminergic status in the striatum, and the increase in D3R expression after chronic treatment with l-DOPA, in sharp contrast with D3R agonists (see above), could be another marker of the striatal hypodopaminergic state. The hypodopaminergic state is also consistent with the modest efficacy of combining l-DOPA with D2R/D3R agonists, which otherwise would have been meaningless if the DARs were already stimulated by l-DOPA-derived DA. As explained elsewhere, l-DOPA promotes a hyperdopaminergic trait in extrastriatal regions, since serotonergic terminals display higher densities compared to DA terminals in extrastriatal territories [16].

Since most molecular studies have been conducted at the level of the striatum, it is difficult to transpose the molecular data obtained at this level to other brain regions as far as DA transmission is involved. LID has been associated with serotonergic neurons [15,281,282]. In variance with past hypotheses, the level of DA tone per se does not seem to play a role in LID [16,85]. Meanwhile, dyskinetic monkeys treated with l-DOPA for 6 months did not show an increase in striatal DA extracellular levels after a challenge dose of l-DOPA [112], while chronic treatment with l-DOPA reduced the ability of l-DOPA to enhance DA extracellular levels in 6-OHDA rats [283,284,285]. Finally, LID can be produced after chronic treatment with l-DOPA at doses that poorly increase the extracellular levels of DA in a situation where DA levels are dramatically magnified due to the absence of DAT [16]. The questioning of the role of striatal DA in LID is also necessary for the therapeutic benefit of l-DOPA. As reported above, 5-HT1AR agonists, such as buspirone, that reduce the activity of serotonergic neurons, are being tested in the clinic to further their potential use as anti-dyskinetic agents in LID, as initially reported in 1994 [233].

As the levels of DA induced by l-DOPA are not predictive of the behavioral outcomes, the study of DARs could be considered as delineated above. Nonetheless, this study cannot only address the striatum and should include the structures of the pyramidal tract (notably the spinal cord) together with brainstem nuclei [286]. D3Rs are indeed expressed in cortical areas as well as in the spinal cord. In the spinal cord, DARs may play various roles, even though DA innervation is extremely low, while in the cortex, they may act on cognition [287,288]. The ambivalent responses of D3R agonists and antagonists depending on the study could reflect the participation of several loci in the behavioral responses, including the striatum. A better understanding of their role outside the striatum is important to correctly address the pharmacology of D3R in PD.

## 11. Conclusions

Even though the D2R and D3R share similar pharmacological profiles, there are some mechanistic differences in their signaling pathways. Indeed, while D2R-induced ERK activation is Giα-dependent pathway-mediated, D3R-induced ERK activation involves a mechanism dependent on Gβγ [289]. Therefore, it is appropriate to consider that the D3R could be regarded as a potential target for anti-parkinsonian drugs [290]. Mesolimbic and striatal D2R blockade results in disturbed motor behavior, while selective D3R inactivation improves motor behavior [3,79,291,292]. Indeed, increasing evidence suggests a pivotal role of the D3R in PD as a therapeutic target. However, the D3R-induced modulation of expression (PD-like symptoms and LID resulting in down- and upregulation of D3R expression), localization, and regional functions during chronic exposure to l-DOPA treatment, resulting in both beneficial motor effects and impaired motor functions, illustrates the complexities of delineating the neurobiological mechanism of D3Rs in PD. Chronic l-DOPA treatment triggers neuroplasticity that aberrantly alters BG circuitry, leading to abnormal motor effects as expressed in dyskinesia [274].

Future studies should identify all the key players in D3R signaling and their putative functional involvement in mediating LID, and closely examine whether they are contributing to the development of the LID, or if they are, in fact, a consequence of LID. Overall, there is a substantial amount of evidence supporting the role of D1Rs and D3Rs in LID involving cooperative striatal interaction as D1R–D3R heteromers [274].

The importance of oligomerization in DARs for developing strengthening links between the receptor subtypes exhibiting emergent functional properties may be relevant to provide new pharmacological approaches targeting the functional dimeric rearrangements of D3R (e.g., D3R–D1R). Indeed, while the involvement of D1 and D3 receptors in LID is well documented, the pharmacological approach of antagonizing these receptors has proven to be difficult to deploy [152,293,294]. However, the renewed interest in these receptors arises from the finding that chronic l-DOPA treatment can trigger the expression of D1R–D3R heteromeric interactions in the dorsal striatum. Moreover, a better understanding of the mechanism of action of l-DOPA could change the views centered in the striatum toward integrated mechanisms from the cortex to the spinal cord, with the D3R being expressed at all the stages.

## Figures and Tables

**Table 1:** Some D3 receptor-selective agonists and antagonists according to their Ki and their selectivity over D2 receptors.

D3 Receptor Agonists			
Ligand	Ki D3R	Selectivity over D2 Receptors	References
DA	3.9 nM	DR2/DR3 = 0.4	[154]
Bromocriptine	0.5–5 nM		[155]
Cabergoline	<0.5 nM		[156]
CJ-1037			
D-264	0.92 nM	DR2/DR3 = 253	[157]
D-440		DR2/DR3 = 583.2	[158]
Quinpirole	0.96 nM	DR2/DR3 = 133	[159,160]
Pergolide	0.5–5 nM		[155]
Pramipexole	0.5–8.5 nM	DR2/DR3 = 253	[161,162,163]
R-7-OH-DPAT		DR2/DR3 = 26	[164]
Ropinirole	69 nM	DR2/DR3 = 8.3	
Lisuride	1.08 nM	DR2/DR3 = 2.5	[165,166]
**D3 Receptor Antagonists**			
**Ligand**	**Ki D3R**	**Selectivity over D2 Receptors**	**References**
FAUC 365	0.50 nM	DR2/DR3 = 200	[167]
BP 897	0.92 nM	DR2/D3R = 66	[168,169]
GMC1111	1.4 nM	DR2/DR3 = 19	[170]
GR103691			
GR218231	1.3 nM	DR2/DR3 = 380	[159,171]
Nemonapride	<0.5 nM		[172]
NGB 2849	0.9 nM	DR2/DR3 = 290	[173]
PD128907	5–50 nM		[174]
Raclopride	0.5–5 nM		[172]
S-5-OH-DPAT		DR2/DR3 = 60	[164]
S-nafadotride	0.3 nM	DR2/DR3 = 10	[175]
S14297	13 nM	DR2/DR3 = 23	[176,177]
S33084	0.32 nM	DR2/DR3 = 100	[159,178,179]
SB 277011	10 nM	DR2/DR3 = 100	[180]
Spiperone	0.5–5 nM		[172]
ST 198	12 nM	DR2/DR3 = 65	[2,181]
U99194A	31 nM	DR2/DR3 = 32	[182]

## Data Availability

Not applicable.
